# Survival influence of gender on 42,345 patients with gastric cardia adenocarcinoma

**DOI:** 10.1007/s00432-022-04470-8

**Published:** 2022-11-14

**Authors:** Rui Hua Xu, Xue Ke Zhao, Xin Song, Ling Ling Lei, Kan Zhong, Wen Li Han, Ran Wang, Qi De Bao, Jing Feng Hu, Meng Xia Wei, Jia Jia Ji, Liu Yu Li, Zong Min Fan, Xue Na Han, Bei Li, Yuan Ze Yang, Lin Sun, Jia Li, Miao Miao Yang, Xing Song Li, Duo You, He Lin Bai, Jia Xin Qiao, Ye Zhen Xie, Fu You Zhou, Xue Min Li, Ai Li Li, Li Dong Wang

**Affiliations:** 1 State Key Laboratory of Esophageal Cancer Prevention and Treatment and Henan Key Laboratory for Esophageal Cancer Research of The First Affiliated Hospital, Zhengzhou University, Zhengzhou, 450052 Henan Province People’s Republic of China; 2 Department of Oncology, Anyang District Hospital, Anyang, 450052 Henan Province People’s Republic of China; 3 Department of Medical Oncology, Henan Cancer Hospital, Affiliated Cancer Hospital of Zhengzhou University, Zhengzhou, 450008 Henan Province People’s Republic of China; 4 Department of Thoracic Surgery, Anyang Cancer Hospital, Anyang, 455000 Henan Province People’s Republic of China; 5 Department of Pathology, Ci County People’s Hospital, Handan, 056599 Hebei Province People’s Republic of China; 6 Department of Oncology, Linzhou Cancer Hospital, Anyang, 456550 Henan Province People’s Republic of China

**Keywords:** Gender, Prognosis, Gastric cardia adenocarcinoma, Clinicopathological characteristics

## Abstract

**Purpose:**

Some studies indicated that gender is associated with prognostic of cancer, However, currently the prognostic value of gender for gastric cardia adenocarcinoma (GCA) survival is unclear. The aim of our study is to reveal the influence of gender on the prognosis of patients with GCA.

**Patients and methods:**

A total of 42,345 cases Chinese GCA patients were enrolled from our previously established GCA and esophageal cancer databases. The clinicopathological characteristics were retrieved from medical records in hospital. The follow-up was performed through letter, telephone or home interview. Among GCA patients, there were 32,544 (76.9%) male patients with the median age 62 years (range 17–97) and 9,801 (23.1%) female patients with the median age 61 years (range 17–95 years). The Chi-square test and Kaplan–Meier method were used to compare the continuous variables and survival. Cox proportional hazards model was used for competing risk analyses, hazard ratios (HRs) and 95% confidence intervals (CIs) were evaluated.

**Results:**

Men had shorter GCA-specific survival than women by multivariate analysis (HR 1.114; 95% CI 1.061 to 1.169; *P* < 0.001). Whether premenopausal, perimenopausal or postmenopausal, the survival of women was better than that of men (premenopausal *vs.* male, *P* < 0.001; perimenopausal *vs.* male, *P* < 0.001; postmenopausal *vs.* male, *P* = 0.035). It was worth noting that in patients with stages I, II, III, and IV, female patients survive longer than male patients (*P* = 0.049; *P* = 0.011; *P* < 0.001; *P* = 0.044, respectively).

**Conclusion:**

Gender is an independent prognostic factor for patients with GCA. In comparison with men, women have a significantly better outcome. Smoking and drinking may be protective factors for male GCA patients.

## Introduction

Gastric cardia adenocarcinoma (GCA) is one of the most common digestive system malignant tumors and often concurrently occurs with esophageal squamous cell carcinoma (ESCC) in China (Tran et al. 2005). GCA epidemiologically shares a very similar geographic distribution with ESCC, in China, especially in Linzhou, Henan Province, north China, the highest incidence area of ESCC in the world (Wang et al. 1997, 1994). GCA bears many similarities to ESCC in terms of common geographic distribution and environmental risk factors (Zhang et al. 2017a, b). There is evidence that GCA differs from cancer of the rest of the stomach in terms of time, trend, risk factors, and histopathogenesis. More than half of GCA cases occur in China (Colquhoun et al., 2015).

In China, both GCA and ESCC are more frequent in men than in women. Although this may represent various tumor-specific environmental exposures between men and women (such as tobacco, alcohol), growing studies suggest hormonal influences (Wang et al. 2011; Zhang et al. 2017a, b; Derakhshan et al. 2009). Gender differences affect GCA incidence, yet the significance of gender as an independent prognostic marker is unclear. The potential role of patients’ gender is often left unexplored and even under-reported, in cancer research. Some studies have shown higher survival for women, compared to men, such as colorectal cancer (Kotake et al. 2016; Hendifar et al. 2009), lung cancer (Ulas et al. 2015; Kinoshita et al. 2017), and melanoma (Joosse et al. 2012). In contrast, women had lower survival from cancers of the bladder, renal pelvis, and ureter (Afshar et al. 2018a, b; Patel et al. 2015). So far, few of the studies have definitely clarified gender differences in cancer survival. In this study, we investigated gender differences in GCA survival using 42,345 patients with GCA to clarify whether gender is an independent prognostic factor for GCA and whether the influence of gender on the survival of GCA is changed by other clinicopathological characteristics (age, high/low incidence area, stage, family history, drinking, smoking, and type of treatment). This study can provide reference value for clinical diagnosis, treatment and individualized prevention of GCA.

## Patients and methods

### Patients

All the patients were enrolled from the 500,000 esophageal and GCA databases (1973–2021) established by the State Key Laboratory for Esophageal Cancer Prevention and Treatment and Henan Key Laboratory for Esophageal Cancer Research of the First Affiliated Hospital of Zhengzhou University (Zhengzhou, China). Case inclusion criteria: (1) detailed place of origin and current address; (2) postoperative pathology confirmed as GCA; (3) complete pathological information. The patient information collected in this study included age, high/low incidence area, stage, family history, drinking, smoking, and type of treatment.

### Survival follow-up

The diagnosis time of the patient refers to the time of esophageal squamous cell carcinoma diagnosed by histopathology; the survival time is the time from diagnosis to death (end event) or the last follow-up. The initial time of follow-up was the time of pathological diagnosis of the patients. The patients were followed up by letter, telephone, village doctor inquiry and household survey, and the patients were followed up every 3 months in the first year and every year from the second year.

### Check and supplement of clinicopathological information

All clinical information for each patient was collected and digitalized based on in-patient medical records, including gender, age at diagnosis, high and low incidence areas, family history (two or more esophageal cancer patients in the same family within consecutive three generations), cigarette smoking, alcohol consumption, histopathology and treatment procedures. Pathological diagnosis was based on the medical record for each patient. All patients with esophagectomy were staged according to the 2002 American Joint Committee on Cancer (AJCC) tumor node metastasis staging system for esophageal cancer (Greene et al. 2002).

### Division standard of high and low incidence areas

The division of high and low incidence areas is based on the book “esophageal Cancer”. According to the results of epidemiological investigation of esophageal cancer in China, the age and mortality are adjusted. The areas with a mortality rate of more than 60/100,000 are high incidence areas, and the rest are low incidence areas (Henan Medical College 1983).

### Menopausal status

We did not have information on menopausal status of the women at diagnosis of GCA, and so age at cancer was used as a surrogate for menopausal status: age ≤ 45 years, premenopausal women; 46 ≥ age ≤ 55 years, perimenopausal women; age ≥ 56, postmenopausal women (Mellemkjaer et al. 2006).

### Statistical analysis

SPSS21.0 was used for statistical analysis, *T* test was used to compare the value of continuous variables between study groups. Chi-square (for more than two groups) or Fisher’s exact test (for two groups) were used to compare the value of categorical variables between study groups. Survival time was calculated according to the year. The survival curves of patients with different clinicopathological characteristics were drawn by Kaplan–Meier method and tested by Log rank. The main factors affecting survival were analyzed by multi-factor Cox proportional hazard regression model.

## Results

### Patient characteristics

This study included a total of 42,345 cases Chinese GCA patients, diagnosed from 1973 to 2021. The proportion of patients diagnosed in 1973 to 1982, 1983 to 1992, and 1993 to 2002, 2003 to 2012, 2013 to 2021 was 2.5%, 9.4%, 17.5%, 55% and 15.6%, respectively. Ratio of men to women was 3.3:1. The men median age was 62 years (range 17–97 years), average age was 61.40 ± 9.25. The women median age was 61 years (range 17–95 years), average age was 60.64 ± 9.58.

### Clinicopathologic characteristics of men and women

Table 1 shows the comparisons between men and women by age, smoking, drinking, high/low incidence area, family history, pathological stage, and type of treatment. There was no difference between these 2 groups in the percentage of family history (*P* = 0.63), pathological stage (*P* = 0.28) and type of treatment (*P* = 0.98). However, it showed that the men group had a significantly difference proportion of age and high/low incidence area (*P* < 0.01), higher proportion of smoking and drinking (respectively, 61.5% *vs.* 4.1%, *P* < 0.01; 38.2% *vs.* 2.8%, *P* < 0.01).

**Table 1:** Clinicopathological characteristics by gender in patients with GCA, *n* (%)

Characteristic	Male	Female	*P*
Age, years			< 0.01
< 40	439 (1.3)	198 (2.0)	
40−	2,699 (8.3)	934 (9.5)	
50−	9,776 (30.0)	3,120 (31.8)	
60−	13,394 (41.2)	3,812 (38.9)	
70−	5,583 (17.2)	1,578 (16.1)	
80 +	653 (2.0)	159 (1.6)	
Total	32,544 (76.9)	9,801 (23.1)	
Smoking			< 0.01
Yes	11,969 (61.5)	240 (4.1)	
No	7,493 (38.5)	5,662 (95.9)	
Total	19,462 (76.7)	5,902 (23.3)	
Drinking			
Yes	7,298 (38.2)	167 (2.8)	< 0.01
No	11,807 (61.8)	5,717 (97.2)	
Total	19,105 (76.5)	5,884 (23.5)	
Regions*			
HIA	21,442 (66.1)	7,248 (74.3)	< 0.01
LIA	10,985 (33.9)	2,506 (25.7)	
Total	32,427 (76.9)	9,754 (23.1)	
Family history#			
PFH	5,760 (27.3)	1,735 (27.6)	0.63
NFH	15,371 (72.7)	4,558 (72.4)	
Total	21,131 (77.1)	6,593 (22.9)	
Pathological stage			
0 + I	988 (4.8)	319 (5.4)	0.28
II	1,862 (9.0)	533 (9.0)	
III	16,577 (79.8)	4,716 (79.4)	
IV	1,337 (6.4)	374 (6.3)	
Total	20,764 (77.8)	5,942 (22.2)	
Type of treatment**			
Surgery	25,728 (96.5)	7,485 (96.5)	0.98
RCT	937 (3.5)	273 (3.5)	
Total	26,665 (77.5)	7,758 (22.5)	

*HIA, high incidence area; LIA, low incidence area (the areas with an incidence rate of more than 60/100,000 are high incidence areas, and the rest are low incidence areas)

^#^PFH, positive family history; NFH, negative family history; ** RCT, radiochemotherapy

### Cox regression analyses

**Fig. 1:**
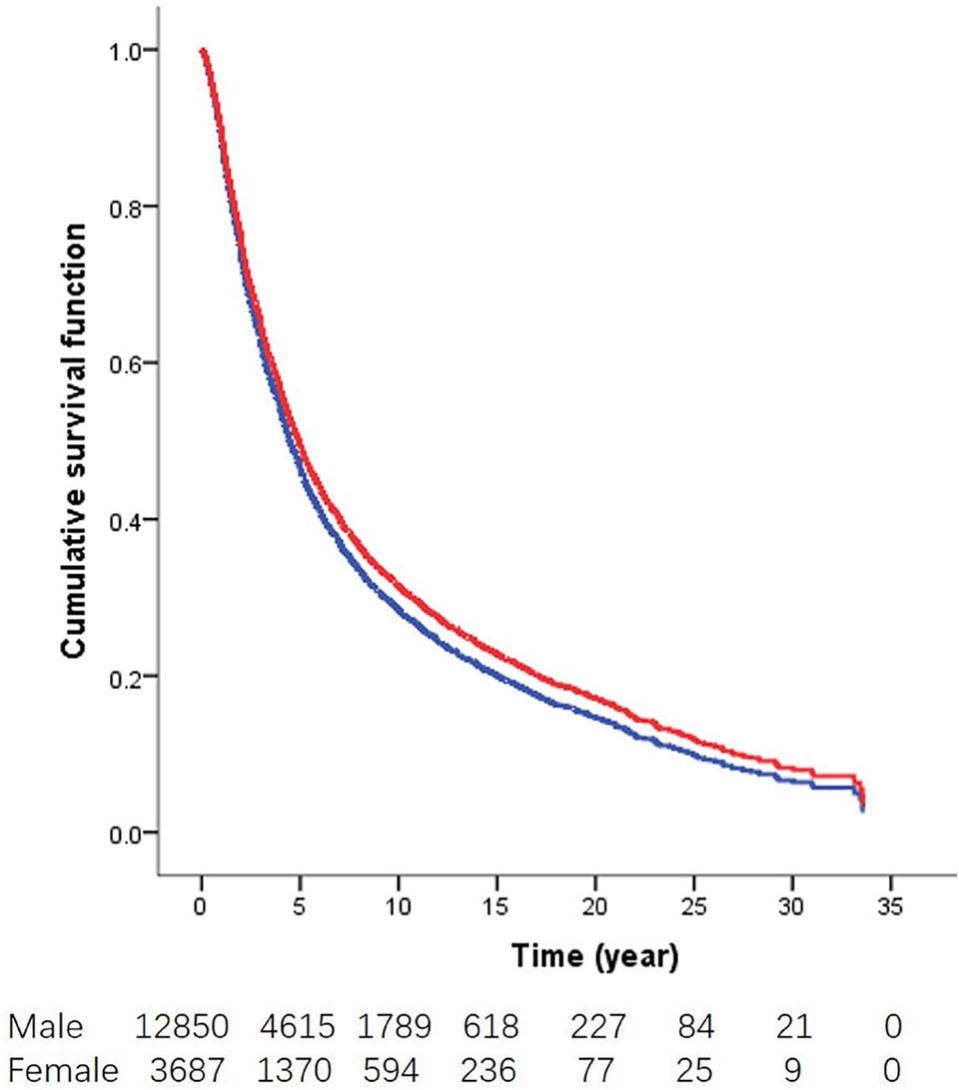
Adjusted survival curves of GCA patients by gender

Based on survival in competing risks regression model, including all variables in Table 2 and smoking status, gender (*P* < 0.001), age (*P* < 0.001), high/low incidence area (*P* < 0.001), family history (*P* < 0.001), drinking (*P* < 0.001), pathological stage (*P* < 0.001) and type of treatment (*P* < 0.001) were independent factors for the survival of GCA. Male (HR 1.114; 95% CI 1.061–1.169; *P* < 0.001), ≥ 60 years old (HR 1.114; 95% CI 1.061–1.169; *P* < 0.001), non-drinking(HR 1.074; 95% CI 1.023–1.127; *P* = 0.004), negative family history(HR 1.090; 95% CI 1.044–1.138; *P* < 0.001), II stage(HR 1.227; 95% CI 1.061–1.419; *P* = 0.006), III stage(HR 2.627, 95% CI 2.000–2.569; *P* < 0.001), IV stage(HR 4.172; 95% CI 3.631–4.794; *P* < 0.001), radiochemotherapy (HR 1.609; 95% CI 1.204–2.151; *P* < 0.001) were the risk factors of GCA patients, and the low incidence area (HR 0.905; 95% CI 0.867–0.944; *P* < 0.001) was the protective factors of GCA patients (Table 2). In addition, adjusted survival curves of GCA patients by gender showed women had longer survival time than men (*P* < 0.001; Fig. 1).

**Table 2:** Multivariate analysis in patients with GCA

Characteristic	No. of patients	No. of death	HR(95% CI)	*P*
Gender				< 0.001
Female	3,687	2,283		
Male	12,850	8,292	1.114(1.061–1.169)	0.000
Age, years				< 0.001
< 60	6,158	3,736		
≥ 60	10,379	6,839	1.364(1.311–1.420)	0.000
Regions*				< 0.001
HIA	11,298	7,443		
LIA	5,239	3,132	0.905(0.867–0.944)	0.000
Drinking				0.004
Yes	5,167	3,121		
No	11,370	7,454	1.074(1.023–1.127)	0.004
Family history^#^				< 0.001
PFH	4,798	2,907		
NFH	11,739	7,668	1.090(1.044–1.138)	0.000
Pathological stage				< 0.001
0 + I	843	253		
II	1,376	651	1.277(1.061–1.419)	0.006
III	13,109	8,724	2.627(2.000–2.569)	0.000
IV	1,209	947	4.172(3.631–4.794)	0.000
Type of treatment**				0.001
Surgery	16,468	10,529		
RCT	69	46	1.609(1.204–2.151)	0.001

*HR* hazard ratio, *HIA* high incidence area; *LIA* low incidence area

*Based on survival in competing risks regression model, including all variables in the table and smoking

**RCT radiochemotherapy

^#^*PFH* positive family history; *NFH* negative family history

### Gender, menopausal status and survival of GCA

**Fig. 2:**
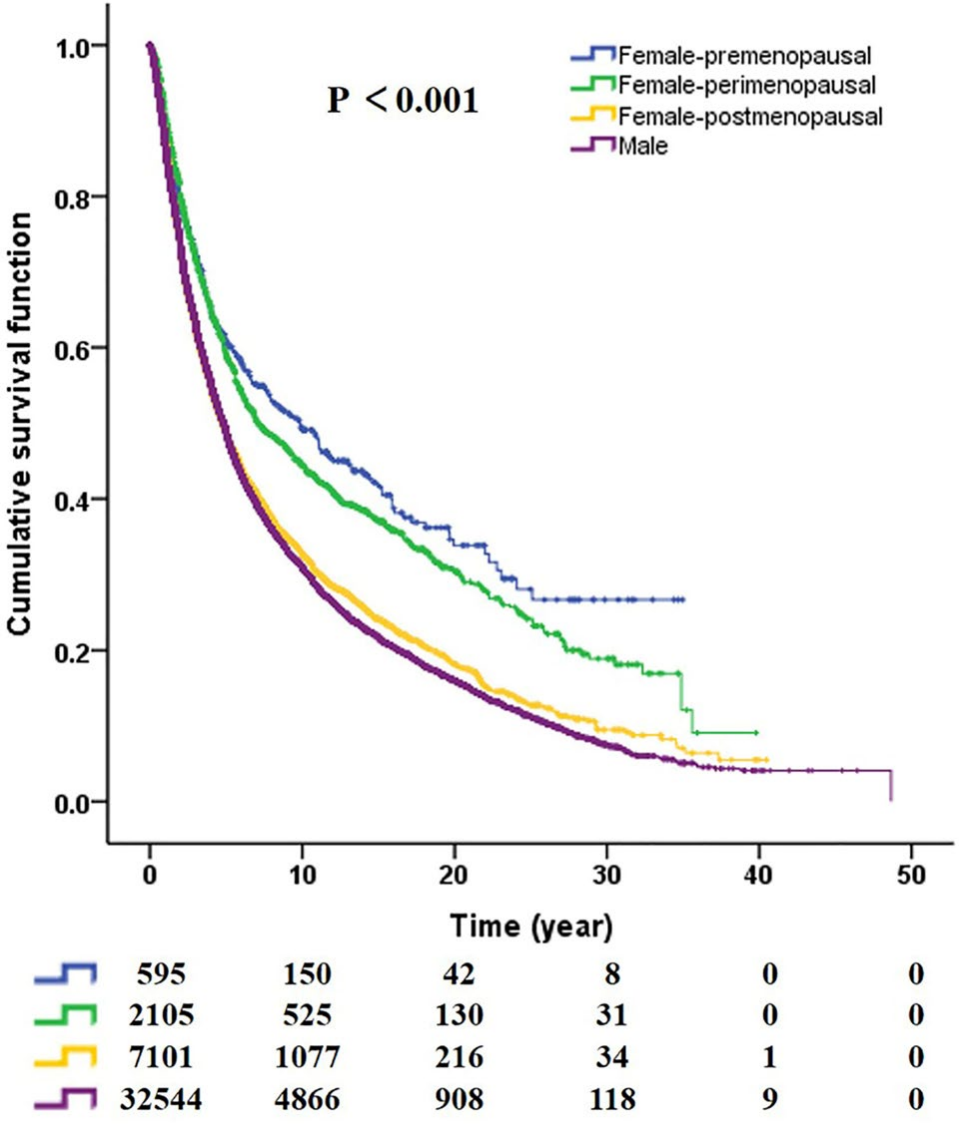
Survival curves of women with different menopausal states and men (premenopausal vs. male, *p* < 0.001; perimenopausal vs. male, *p* < 0.001; postmenopausal vs. male, *p* = 0.035)

Whether premenopausal, perimenopausal or postmenopausal, the survival of women was better than that of men (premenopausal vs. male, *P* < 0.001; perimenopausal vs. male, *P* < 0.001; postmenopausal vs. male, *P* = 0.035; Fig. 2).

### Gender, pathological stage and survival of GCA

**Fig. 3:**
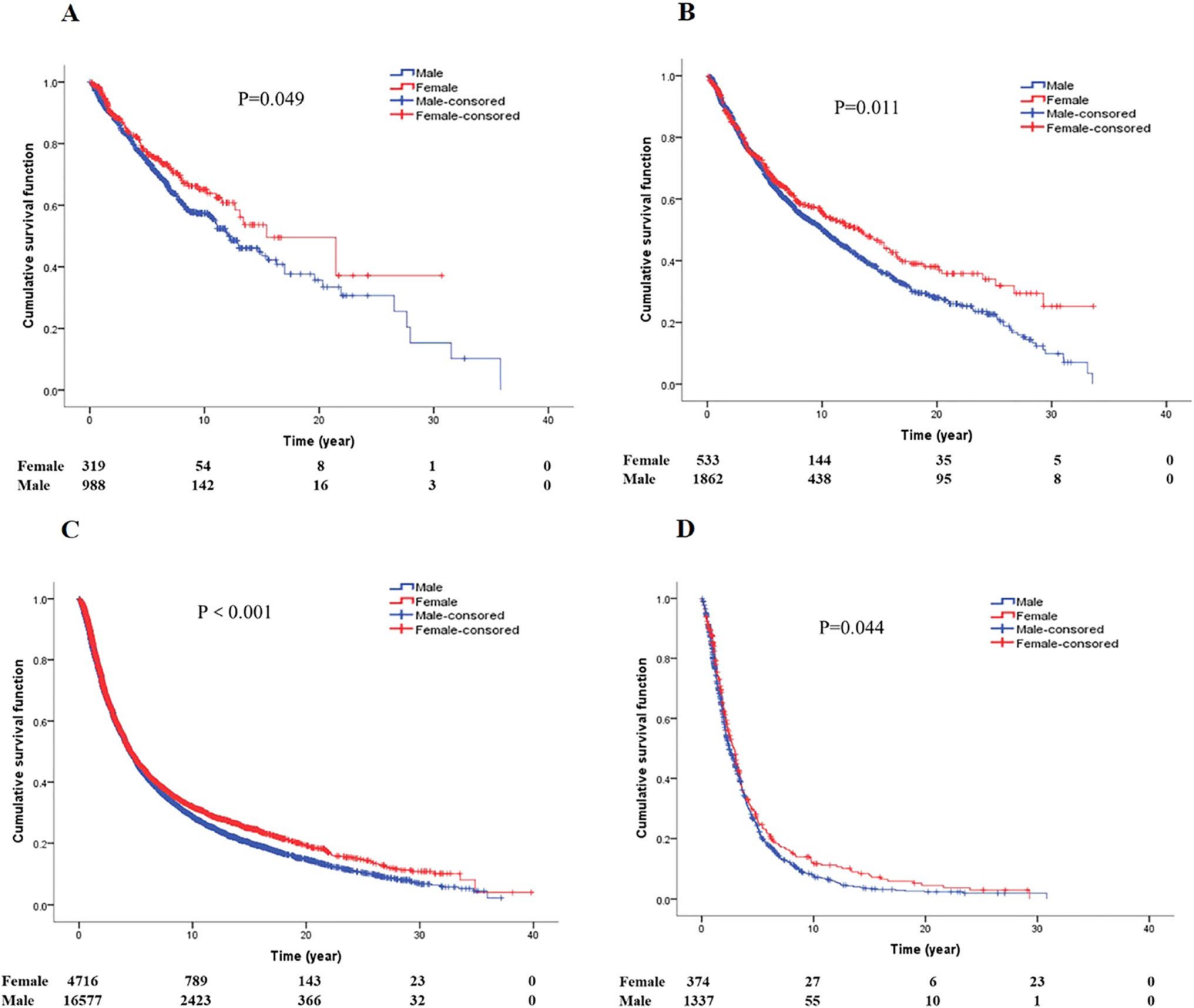
Survival curves of GCA patients by stage (**A** survival curves of gender by stage 0 + I. **B** Survival curves of gender by stage II. **C** Survival curves of gender by stage III. **D** Survival curves of gender by stage IV)

Survival analysis between gender and pathological stage for GCA patients (Fig. 3). It was worth noting that in patients with stages I, II, III, and IV, female patients survive longer than male patients (*P* = 0.049; *P* = 0.011; *P* < 0.001; *P* = 0.044; respectively).

### Gender, age and survival of GCA

**Fig. 4:**
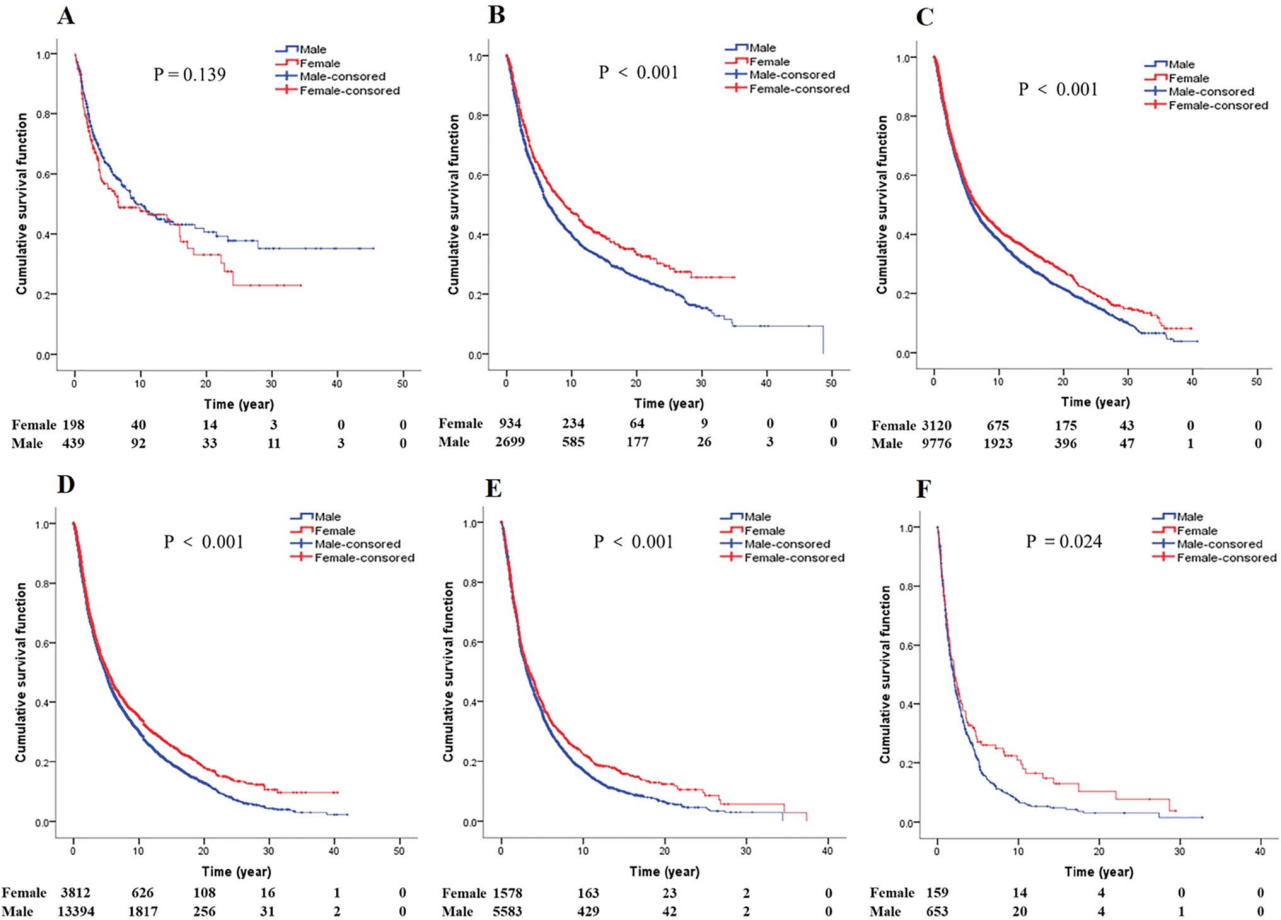
Age-stratified survival curves of gender for GCA patients (**A** Survival curves of gender for GCA patients less than 40 years old. **B** Survival curves of GCA patients aged 40–49. **C** Survival curves of GCA patients aged 50–59. **D** Survival curves of GCA patients aged 60–69. **E** Survival curves of GCA patients aged 70–79. **F** Survival curves of GCA patients aged older than 79)

Survival analysis of gender in different age groups (Fig. 4). In the younger age groups (< 30 years old, 30–40 years old), there was no significant difference in the survival rate between genders (*P* = 0.223; *P* = 0.212). However, in the older age groups (40–49 years old; 50–59 years old; 60–69 years old; 70–79 years old; > 80 years old), female patients lived longer than male patients (respectively, *P* < 0.001; *P* < 0.001; *P* < 0.001; *P* < 0.001; *P* = 0.024).

### Gender, high/low incidence area and survival of GCA

**Fig. 5:**
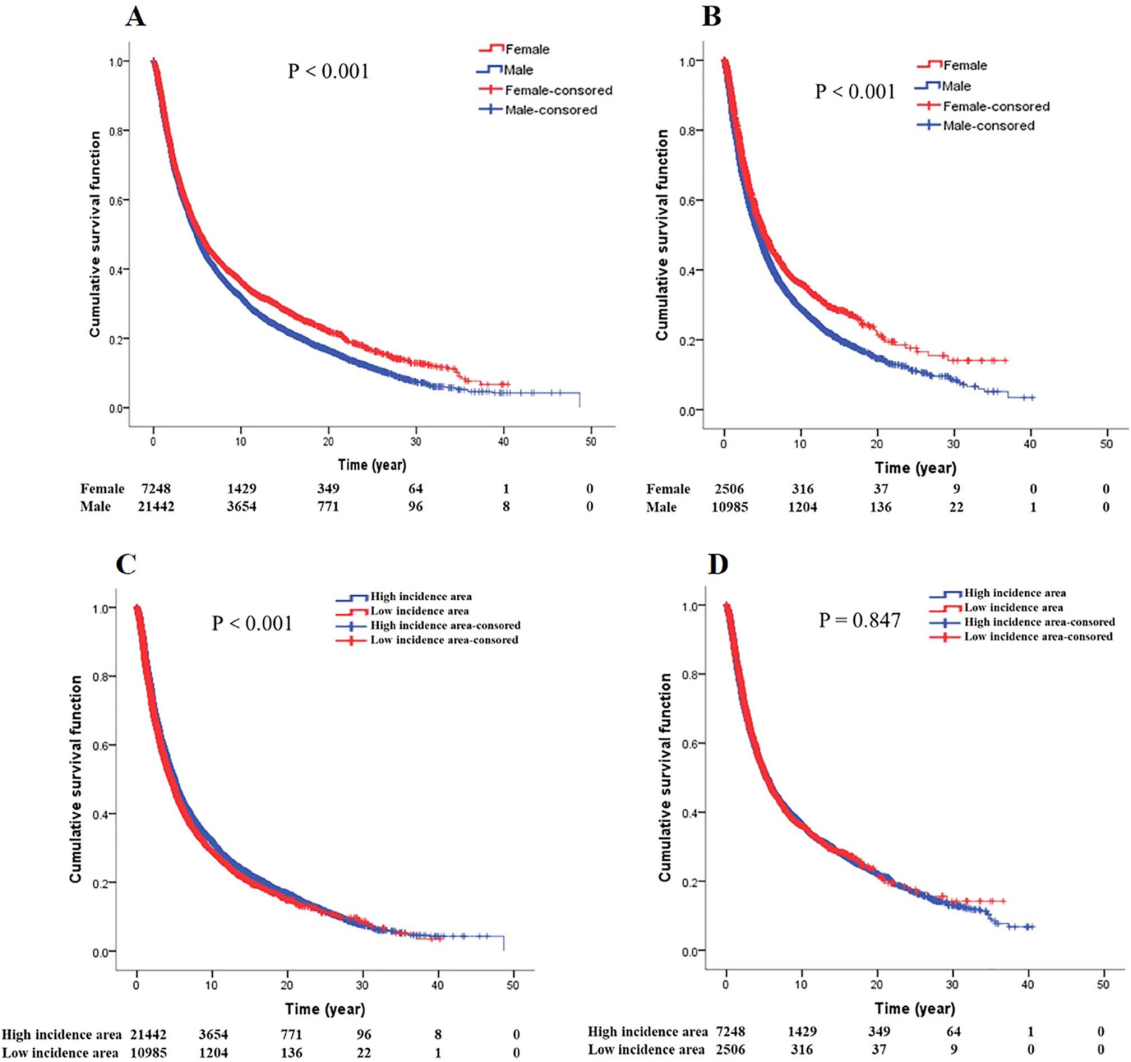
Gender and high/low incidence area (**A** Survival curves of gender in high incidence area. **B** Survival curves of gender in low incidence area. **C** Survival curves of high/low incidence area in male GCA patients. **D** Survival curves of high/low incidence area in female GCA patients)

Survival analysis between gender and high/low incidence area for GCA patients (Fig. 5). Regardless of whether GCA patients were in high or low incidence areas, male patients lived worse than female patients (*P* < 0.001; *P* < 0.001, respectively). In male patients, the survival in the high incidence area was better than that in the low incidence area (*P* < 0.001); however, in female patients, there was no difference in survival between the high incidence area and the low incidence area (*P* = 0.847).

### Gender, family history and survival of GCA

**Fig. 6:**
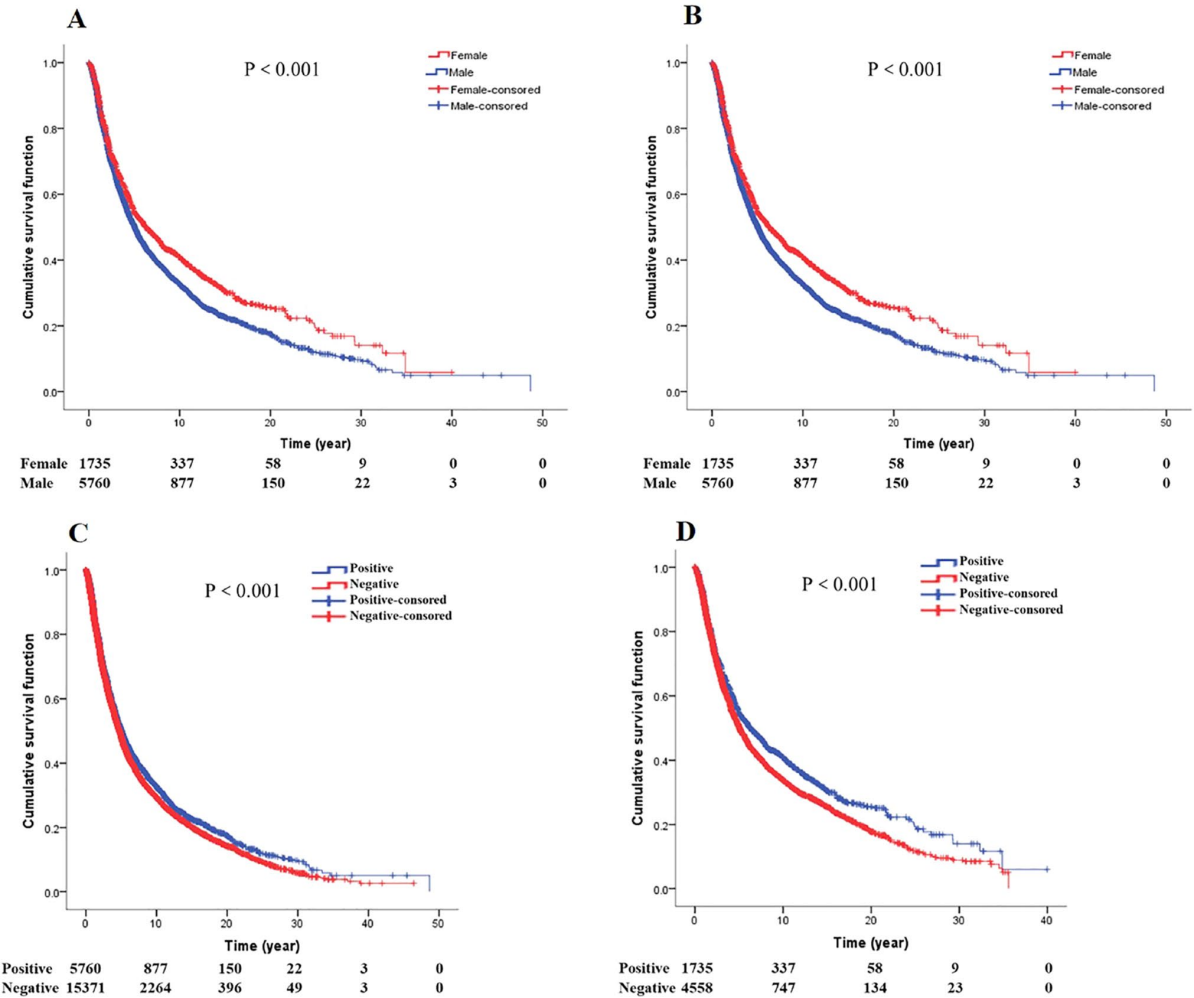
Gender and family history (**A** survival curves of gender by family history positive. **B** survival curves of gender by family history negative. **C** Survival curves of family history in male GCA patients. **D** Survival curves of family history in female GCA patients)

Survival analysis between gender and family history for GCA patients (Fig. 6). Regardless of whether the family history of GCA patients is positive or negative, women live longer than men (*P* < 0.001; *P* < 0.001, respectively). In addition, both in male and female patients, the survival of patients with positive family history was better than that of patients with negative family history (*P* < 0.001; *P* < 0.001, respectively).

### Gender, smoking and survival of GCA

**Fig. 7:**
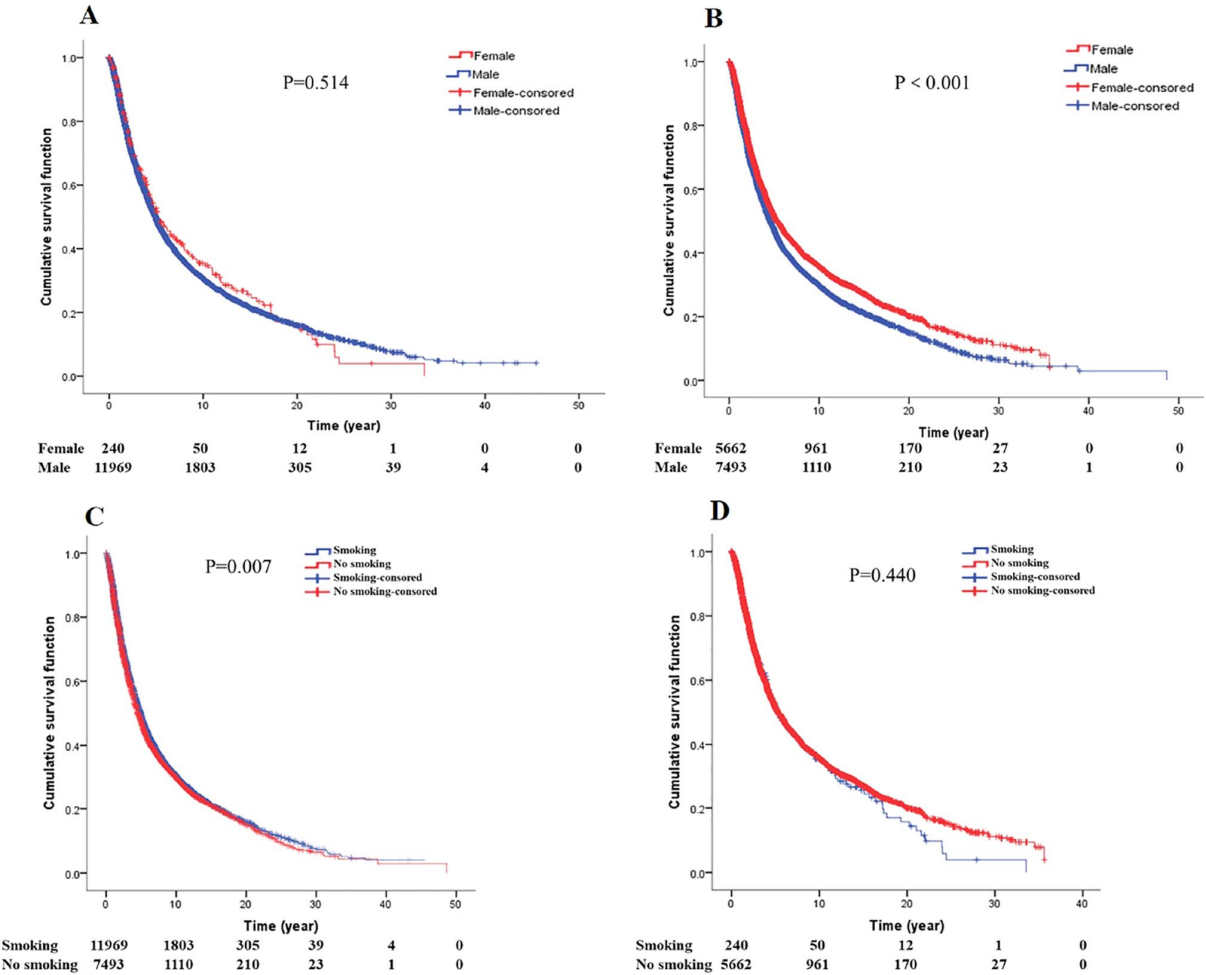
Gender and smoking (**A** survival curves of gender by smoking. **B** Survival curves of gender by no smoking. **C** Survival curves of smoking in male GCA patients. **D** Survival curves of smoking in female GCA patients)

Survival analysis between gender and smoking for GCA patients (Fig. 7). In smoking GCA patients, there was no difference in survival between male patients and female patients (*P* = 0.514). In non-smoking patients, female patients survived longer than male patients (*P* < 0.001). In male patients, the survival of smoking patients was better than that of non-smoking patients (*P* = 0.007), while in female patients, there was no difference in survival between smoking and non-smoking (*P* = 0.440).

### Gender, drinking and survival of GCA

**Fig. 8:**
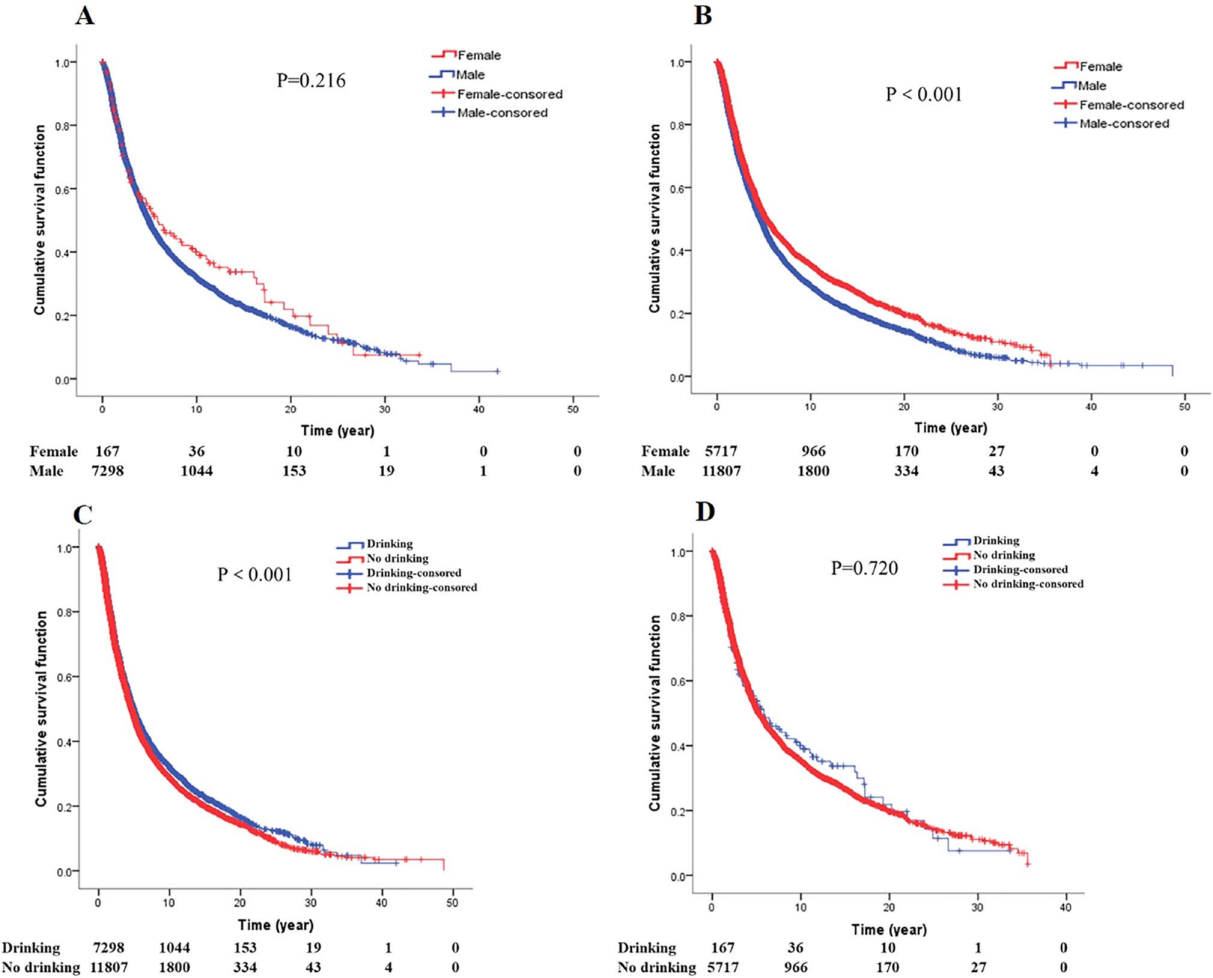
Gender and drinking (**A** survival curves of gender by drinking. **B** Survival curves of gender by no drinking. **C** Survival curves of drinking in male GCA patients. **D** Survival curves of drinking in female GCA patients)

Survival analysis between gender and drinking for GCA patients (Fig. 8). Among the GCA patients who drank alcohol, there was no difference in survival between men and the women (*P* = 0.216). In the patients who did not drink alcohol, the female patients survived longer than the male patients (*P* < 0.001). In male patients, the survival of drinking patients was better than that of non-drinking patients (*P* < 0.001), while in female patients, there was no difference in survival between drinking and non-drinking (*P* = 0.720).

### Gender, treatment and survival of GCA

**Fig. 9:**
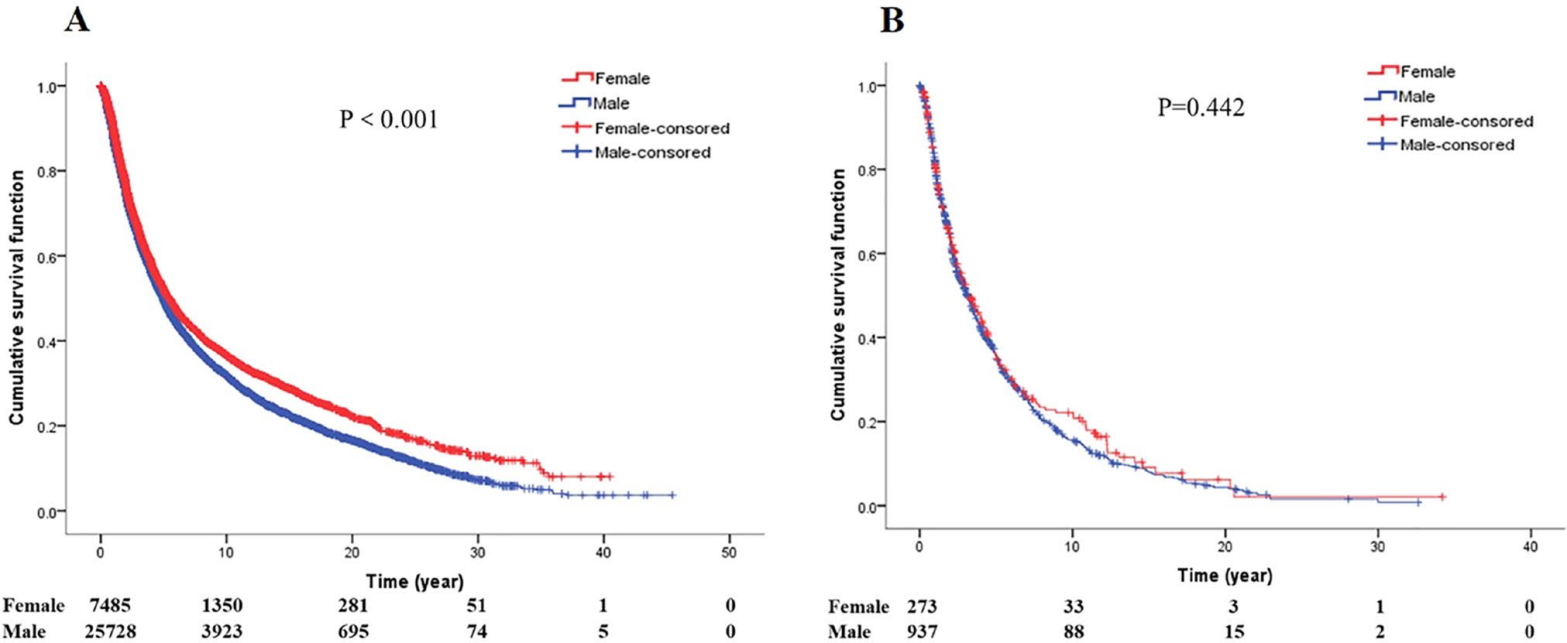
Survival curves of GCA patients by type of treatment (**A** survival curves of gender by surgery. **B** Survival curves of gender by radiochemotherapy)

Survival analysis between gender and treatment for GCA patients (Fig. 9). Male patients with GCA had a shorter survival than female patients when treated with surgery (*P* < 0.001), while there was no difference in male patients' survival compared to female patients when treated with radiotherapy or chemotherapy (*P* = 0.442).

## Discussion

GCA is the most common malignant tumor of digestive tract in Henan Province, China (Gao et al. 2005). Recent decades have seen an alarming increase in the incidence of GCA, while non-cardia gastric adenocarcinoma has decreased (Abdi et al. 2019). At a global scale, total deaths were greater for men than for women (GBD 2017 Causes of Death Collaborators 2018). Some studies have shown that a survival disadvantage for men was observed these cancers: head and neck, esophagus, colorectum, pancreas, lung, bone, melanoma, mesothelioma, kidney, thyroid, and non-Hodgkin lymphoma. In contrast, women had lower survival from cancers of the bladder, renal pelvis, and ureter (Radkiewicz et al. 2017; Afshar et al. 2018a, b). The absolute burden of GCA in China is linked with its poor outcome. As we know, this is the first study to show a statistically significant relationship between gender and the prognosis of GCA patients. In the present study, we showed that gender was an independent prognostic factor for patients with GCA. Women had longer GCA-specific survival than men.

Pathological grade is an independent factor affecting the prognosis of many cancers. In our data, there was no difference in the distribution of pathological grades between men and women. Under the stratification of pathological grade, whether in stage I, II, III or IV, the survival of female patients was dominant, and the difference was statistically significant. This means that pathological grading does not affect the outcome of gender on the prognosis of GCA. Clinically, surgical and non-operative treatment will be selected according to pathological grade. When GCA patients are treated by operation, women survive better than men, but there is no difference in prognosis between female and male patients during radiotherapy and chemotherapy. From the comprehensive point of view of pathological grade and treatment, radiotherapy and chemotherapy may have poor therapeutic effect in female patients with GCA.

In our present study, there was no difference in survival between men and women in the younger age groups (< 40 years), but in the older age groups (40–49 years; 50–59 years; 60–69 years; 70–79 years; > 80 years), the prognosis of women was better than that of men. Overall, age is not the main role of gender in affecting the prognosis of GCA. When the age is less than 40 years old, the reason why there is no difference in survival between male and female patients of GCA needs to be further studied.

The influence of gender on the survival of patients includes factors such as social and environmental hormone differences (exposure to smoking and drinking, etc.). Estrogen is an important gender steroid hormone that plays a significant role in the regulation of many biological functions (Chen et al. 2019). In recent years, researchers have demonstrated that estrogen and its receptors played an important role in gastroesophageal reflux, esophageal cancer, peptic ulcers, gastric cancer (Kim et al. 2016; Kurt et al. 2007). In this study, we did not record the menopausal state of female patients. We divided the menopausal state of female patients into premenopausal, peri-menopausal and postmenopausal by age. It is worth noting that no matter what kind of menopause women are in, they survive better than men, and the difference is statistically significant. This indirectly suggests that estrogen levels may not be the reason why women with GCA survive longer than men.

Zhang et al. found that menopausal status was related to ER expression and ERb positive expression together with ERa negative expression are promising markers for prognosis, which may be provide some theoretical foundation for individualized prevention and endocrinotherapy for female ESCC patients (Zhang et al. 2017a, b). The expression of ER*α* and ER*β* in gastric cancer has been previously demonstrated (Kim et al. 2013). It has been hypothesized that ERs serve an important role in the occurrence and development of gastric cancer (Chandanos et al. 2008). In conclusion, further related studies are needed to in-depth explore the roles and the potential mechanisms of ERa and ERb in GCA survival.

GCA exhibits unevenly geographic prevalence, with the highest incidence in Henan, Hebei and Shanxi Province at the junction of Taihang Mountains, and the incidence gradually decreases around the center. High/low incidence areas represent the important role of environmental factors in the occurrence and development of GCA. In multivariate regression analysis, high/low incidence area is an independent factor on prognosis of patients with GCA. Stratified by high/low incidence area, women had a longer survival than men in both high and low incidence areas. In Henan, China, GCA and esophageal squamous cell carcinoma are called “sister cancers”, which have the same geographical distribution characteristics (Wang et al. 2006). Previous study has revealed that gender is also an independent influencing factor on the prognosis of esophageal cancer, and men live better than women in high-incidence areas, as well as in low-incidence areas (Wang et al. 2010). To sum up, carcinogenic factors such as nitrite and aflatoxin in high incidence areas may not be the main reason why women with GCA live longer than men.

In our study, there was no difference in survival between male and female GCA patients with smoking and drinking habits, while female survival was dominant in non-smoking and non-drinking GCA patients. In our data, the proportion of men who smoke and drink alcohol is much higher than that of women. In addition, we found that among male GCA patients, smokers lived better than non-smokers, and drinkers lived better than non-drinkers. Meta-analysis showed that light alcohol consumption was associated with a reduced risk of cancer mortality of Americans, but alcoholism had serious health consequences and even death (Xi et al. 2017). Kim et al. found that drinking a small amount of alcohol is a protective factor for the prognosis of gastric cancer (Kim et al. 2019). In most gastrointestinal tumor, persistent smokers, especially heavy smokers, have a worse prognosis than never smokers. The prognostic impact of smoking on gender-specific GCA needs to be confirmed in prospective studies. To sum up, smoking and drinking may be a protective factor for the prognosis of male patients, while it may be a risk factor for female patients with GCA. The internal mechanism of gender difference of smoking and drinking on the survival of GCA needs to be further studied. It is still necessary and essential to reduce harmful or heavy drinking and smoking.

In conclusion, our study suggests that gender is an independent and important prognostic factor of GCA patients, and that female patients have a better prognosis than male. We discussed the reasons for women’s superior survival over men from seven aspects (age, pathological grade, menopausal status, treatment mode, high and low incidence area, smoking and drinking, and negative and positive family history), interestingly, smoking and drinking may be protective factors for male GCA patients. The limitations of our study is that it is a retrospective study, and we have not investigated the influence of gender on GCA at the molecular level. In the future, studies of the gender difference in GCA prognosis should focus on the molecular level such as sex hormone receptors, providing new treatments to improve the prognosis of male GCA patients.

## Data Availability

The datasets used and analyzed during the current study are available partly from the corresponding author on reasonable request.
